# Cardiac Tuberculoma Presenting as Sudden Cardiac Death in an Immunocompetent Young Man: A Case Report and Literature Review

**DOI:** 10.1155/2023/2207204

**Published:** 2023-10-04

**Authors:** Alemayehu Shiferaw Lema

**Affiliations:** Department of Forensic Medicine and Toxicology, St. Paul's Hospital Millennium Medical College, Addis Ababa, Ethiopia

## Abstract

Tuberculosis is one of several preventable and curable communicable diseases that is a major cause of morbidity and one of the top ten causes of death worldwide. Cardiac tuberculosis frequently affects the pericardium. Although rare, most cases of tuberculosis involving the myocardium and endocardium present as sudden cardiac death in asymptomatic cases. Information about the myocardial involvement of tuberculosis appears in the literature once in a blue moon. As a result, there is a knowledge gap about cardiac tuberculosis among health professionals. Here, a case of sudden cardiac death from tuberculoma of the right atrium with a secondary thrombus causing bicaval obstruction that is observed in an asymptomatic immunocompetent young man is presented. Furthermore, challenges related to the diagnosis and management of cardiac tuberculosis are discussed, and an overview of the relevant literature is provided to break new ground in early diagnosis and therapy.

## 1. Introduction

Tuberculosis (TB) is one of several preventable and curable communicable diseases that is a major cause of morbidity and one of the top ten causes of death worldwide [1]. According to a World Health Organization (WHO) report, tuberculosis caused 1.4 million deaths globally and remained the top cause of mortality among infectious diseases, even ranking above HIV/AIDS. Ethiopia is among the top 30 countries with a high TB burden for the period 2016–2020 listed more recently by the WHO [2].

Despite being primarily a lung disease, tuberculosis can affect any organ or system of the body. Cardiac involvement in tuberculosis is associated with an unfavorable prognosis [1, 3]. Tuberculosis pericarditis represents the predominant form of cardiac TB [4–6]. The involvement of the myocardium and endocardium is rare, with limited data in the literature, and is available mostly from autopsy series. Although rare, most TB involving the myocardium and endocardium presents as sudden cardiac death (SCD) in asymptomatic cases [5–7].

Information about the myocardial involvement of TB appears in the literature once in a blue moon. As a result, there is a knowledge gap about cardiac TB among health professionals, particularly in resource-poor settings, where TB is prevalent and diagnostic capacities are limited. Despite this enormous burden of TB within the Ethiopian population, this is the first case of TB involving the myocardium and endocardium reported in Ethiopia. Here, a case of sudden cardiac death from tuberculoma of the right atrium with a secondary thrombus causing bicaval obstruction that is observed in an asymptomatic immunocompetent young man is presented. Furthermore, challenges related to the diagnosis and management of cardiac tuberculosis are discussed, and an overview of the relevant literature is provided to break new ground in early diagnosis and therapy.

## 2. Case Report

**Figure 1:**
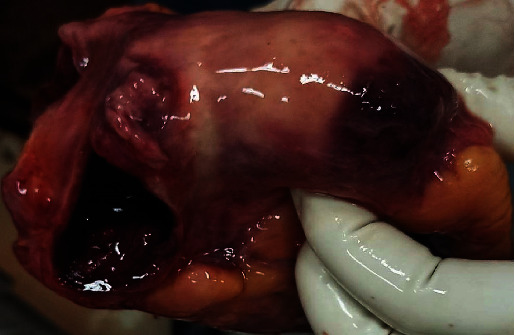
Photograph of the heart showing right atrial tuberculoma with secondary thrombus.

**Figure 2:**
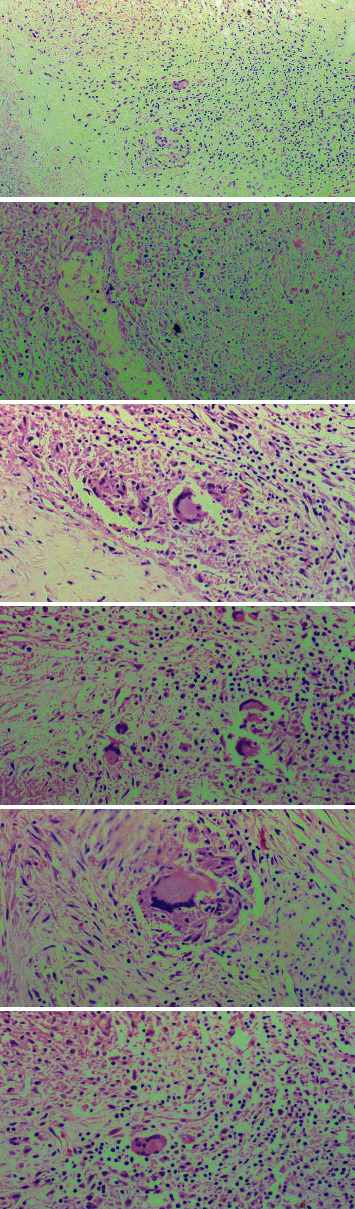
Histological picture of the cardiac mass showing extensive areas of caseous necrosis with numerous well-formed granulomas and Langerhans-type giant cells.

A 31-year-old immunocompetent Ethiopian man was found dead at home inside a washing room for unknown reasons. The spouse reported that he was apparently healthy with no history of prior chronic medical illness or recent medical complaints. There was no history of cardiovascular illness or premature SCD among the relatives. As the cause of death was unknown and unexpected death, police requested a medicolegal autopsy. On external examination, the deceased was well nourished. No evidence of trauma was detected on external or internal examination. Gross examination of the lungs revealed multiple miliary tubercles on both lungs with pleural thickening and adherence to the chest wall. The histology of the lung showed classic granulomas with caseous necrosis, epithelioid cells, and giant Langerhans cells with scattered areas of contraction and fibrosis. Multiple military tubercles were also identified on the liver. The heart was enlarged weighing 570 gms with a moderate increase in epicardial fat. The right atrium was dilated, and there was a globular mass measuring 5.8 cm × 4.9 cm arising from the posterior wall and interatrial septum of the right atrium 0.4 cm above the tricuspid valve, which had almost obliterated the right atrial cavity. It was whitish to yellow in color, firm in consistency, regular borders with a smooth surface, and yellowish brown on the cut section (Figure 1). Thrombus formed over the mass projected further toward the opening of the SVC and IVC into the right atrium, causing inflow obstruction. Histological examination of the heart showed extensive areas of caseous necrosis with numerous well-formed granulomas and Langerhans-type giant cells (Figure 2). Despite the typical histological appearance of a tuberculoma, the Ziehl-Neelsen (ZN) staining of the cardiac mass was negative. Acid-fast bacilli were discovered from the lung sample.

## 3. Discussion

The first case of tuberculous myocarditis was recognized in 1664 by Maurocordat [3]. Myocardial tuberculosis (MTB) is rare, accounting for 0.14% to 2% of cases [1]. The rarity of tuberculous myocarditis has been ascribed to the intrinsic defensive effect of lactic acid produced by myocardial muscle activity [6]. The spread of TB to the myocardium occurs by the hematogenous route (from miliary disease), direct spread, or lymphatic spread from the lymph nodes of the mediastinum [3–6]. TB myocarditis is histologically categorized into three different types: diffuse infiltrative, military, and nodular tubercle. Cardiac tuberculomas (nodular tubercles) are a common form of TB myocarditis and frequently involve the right atrium, as in this case. Nodular tubercles differ significantly in size from a minor nodular lesion to a huge mass, often with typical central caseation [8, 9]. They are frequently sharply demarcated and well circumscribed from the parenchyma that surrounds them. Cardiac tuberculoma occasionally erodes the parenchyma underneath, leading to the formation of an ulcer, which in turn results in the formation of a thrombus and embolism. This may also result in hematogenous spread that causes disseminated TB [6, 9, 10].

The peculiar feature of this case is the disparity between the severity and extent of cardiac tuberculoma found at autopsy and the total absence of prodromal signs and symptoms before death. This is the first report to my knowledge of a large right atrial tuberculoma with complete bicaval obstruction and secondary thrombus in an asymptomatic, immunocompetent individual. Although Horn and Saphir [8] highlighted the rarity of endocardial ulceration in MTB, Kapoor et al. [10] reported the first case of cardiac tuberculoma with disrupted endocardium and secondary thrombus and superior vena cava obstruction syndrome. A case of right atrial tuberculoma in a symptomatic patient with complete superior vena cava obstruction and partial inferior vena cava obstruction and thrombus formation has been reported and treated with anti-TB and anticoagulation therapy that showed significant improvement [9].

The type, location, and size of the lesion determine how MTB presents clinically. Most TB involving the myocardium and endocardium presents as sudden death in asymptomatic cases, as in this case, and is frequently detected at postmortem examinations [4–7]. Cardiac TB accounts for approximately 4% of all deaths related to TB, and most sudden cardiac deaths are caused by TB [4]. TB myocarditis causes SVC obstruction, ventricular obstruction, arrhythmia, valve dysfunction, and severe cardiac failure [1, 3–7, 9–12]. Arrhythmia is a manifestation of TB myocarditis. When a patient is diagnosed with TB in other organs, especially in TB-endemic regions such as Ethiopia, TB myocarditis must be considered in the differential diagnosis [5, 10, 11]. Besides, current globalization requires a comprehensive knowledge of diseases even in their rarest forms. This knowledge is equally important for healthcare professionals residing outside of endemic areas, as they may encounter patients with connections to endemic regions and need to accurately diagnose their conditions. Furthermore, it may present as a ventricular aneurysm, cardiac rupture with tamponade, and consequent death [4, 5, 11]. In a report of 19 cases with cardiac tuberculosis, myocardial involvement of TB was diagnosed before death in a single patient, signifying that the diagnosis of TB myocarditis is challenging and necessitates a high suspicion index [7].

The prompt availability of anti-TB therapy and the advanced diagnostic capacity in developed countries have helped to reduce the burden of TB [4]. Moreover, improvements in cardiovascular imaging, availability of imaging-guided endomyocardial sampling, and molecular biologic capabilities have enabled antemortem detection of TB myocarditis more frequently [4]. Most patients with MTB were found to be of African or Asian origin [4]. Owing to the variable manifestations of myocardial tuberculosis, it is important to treat those patients who have TB and concomitant arrhythmias, flow obstruction, valvular lesions, or heart failure as soon as possible. Early detection of TB myocarditis and immediate initiation of anti-TB therapy for this curable condition are promising strategies for preventing TB-related sudden death. Anti-TB therapy is effective and safe for treating TB myocarditis [4, 11]. Surgical intervention is rarely required when it presents with severe complications such as SVC obstruction, cardiac rupture with a pseudoaneurysm, valvular insufficiency, or life-threatening arrhythmias [11]. Furthermore, complications such as thrombus formation, as in this case, should not be overlooked and require anticoagulant therapy [9].

In the past, patients with intracardiac masses were generally considered to be thrombi or tumors. However, in settings where both TB and HIV/AIDS are common, cardiac tuberculoma must be included in the differential diagnosis. After receiving anti-TB therapy for tuberculosis with myocardial involvement, several studies have shown that the size of myocardial tuberculomas shrinks. As a result, anti-TB treatment is considered a safe and effective method for both diagnosis and management in countries with low resources and high rates of TB [4, 9, 11, 12].

In a typical clinical setting, a tendency for substantial respiratory system examination and investigation in patients suspected of tuberculosis has been excluded in the cold cardiovascular system. This could be because of the low rate, underreporting, and late or missed diagnosis of these cases, which over time have created a knowledge gap among healthcare professionals. To identify patients at risk for sudden cardiac death, particularly those with cardiac structural abnormalities or arrhythmias, routine screening with at least clinical signs and symptoms of cardiac pathologies should be performed in all TB patients. Additionally, all patients with severe, disseminated tuberculosis and those who have cardiac signs/symptoms on routine screening should be investigated with electrocardiography, echocardiography, and even with other cardiovascular imaging as needed.

## 4. Conclusions

Although rare, it is vital to be familiar with the cardiac involvement of tuberculosis that has the potential to cause sudden death, particularly in its miliary form. The diagnosis remains challenging due to the protean manifestations of myocardial tuberculosis. A high suspicion index and increasing recognition of this entity can help us with the timely diagnosis and management of this curable disease. Cardiac tuberculoma should be suspected in patients with intracardiac masses in areas with a high prevalence of tuberculosis, such as Ethiopia.

## Data Availability

Additional data used to support the findings of this study are available from the corresponding author upon request.
